# Application and development of Organ-on-a-Chip technology in cancer therapy

**DOI:** 10.3389/fonc.2025.1643230

**Published:** 2025-09-05

**Authors:** Ling xiao Wang, Shu ling Liu, Ning Wu

**Affiliations:** ^1^ School of Gongli Hospital Medical Technology, University of Shanghai for Science and Technology, Shanghai, China; ^2^ Shanghai Pudong NewArea Gongli Hospital, Shanghai, China; ^3^ Department of Oncology, Shanghai Pudong New Area Gongli Hospital, Shanghai, China

**Keywords:** *in vitro* model, biomedical engineering, drug screening, personalized medicine, organ-on-a-chip, cancer therapy

## Abstract

Cancer therapies are limited by tumor heterogeneity, complex tumor microenvironments (TME), and treatment resistance. Traditional 2D cell cultures cannot replicate tumor 3D architecture and dynamic interactions, reducing clinical relevance. Organoid-on-a-Chip (OoC) technology overcomes these gaps by integrating microfluidics, tissue engineering, and cell biology to create physiologically accurate 3D models. This platform simulates TME dynamics—including vascularization and multi-organ interactions—surpassing static conventional models. Key advancements: (1) Three development phases: basic 3D culture (2009–2015), multi-organ coupling (2016–2020), and clinical translation (2021–present); (2) FDA Modernization Act 2.0 (2022) enabling OoC data as sole preclinical evidence for clinical trials; (3) Patient-derived organoids (PDOs) retaining parental tumors’ features with >87% drug-response accuracy in colorectal cancer. Vascularized tumor chips further study angiogenic dynamics and drug efficacy. While OoC excels in drug screening, toxicity testing, and personalized oncology, challenges persist in simulating systemic immune responses. Advancing multi-organ integration and policy alignment remains critical to replace animal models and advance precision cancer therapy.

## Introduction

Cancer, one of the most common fatal diseases globally, poses a heavy burden on society. Current cancer therapies, while saving numerous lives, have limitations in improving patients’ quality of life and reducing side effects. Enhancing therapeutic efficacy while reducing side effects has become a critical focus in cancer research (1). Traditional radiotherapy and chemotherapy can inhibit tumor growth but inevitably damage normal cells, causing severe side effects. Moreover, intratumoral heterogeneity and the complex tumor microenvironment (Tumor Microenvironment, TME) influence therapeutic responses, and drug resistance further limits treatment efficacy. Conventional *in vitro* models, lacking involvement of live animals or humans, have obvious limitations in fully reproducing the biological characteristics and microenvironment of tumors (2). Traditional 2D cell culture models lack three - dimensional structures and the complexity of the tumor microenvironment. This leads to distorted cell behavior and loss of tumor heterogeneity (2–4). Cell morphology, polarity, and signal transduction are inconsistent with *in vivo* conditions, causing experimental results to deviate from clinical situations and limiting their application in drug screening and personalized medicine (5, 6).

**Figure 1:**
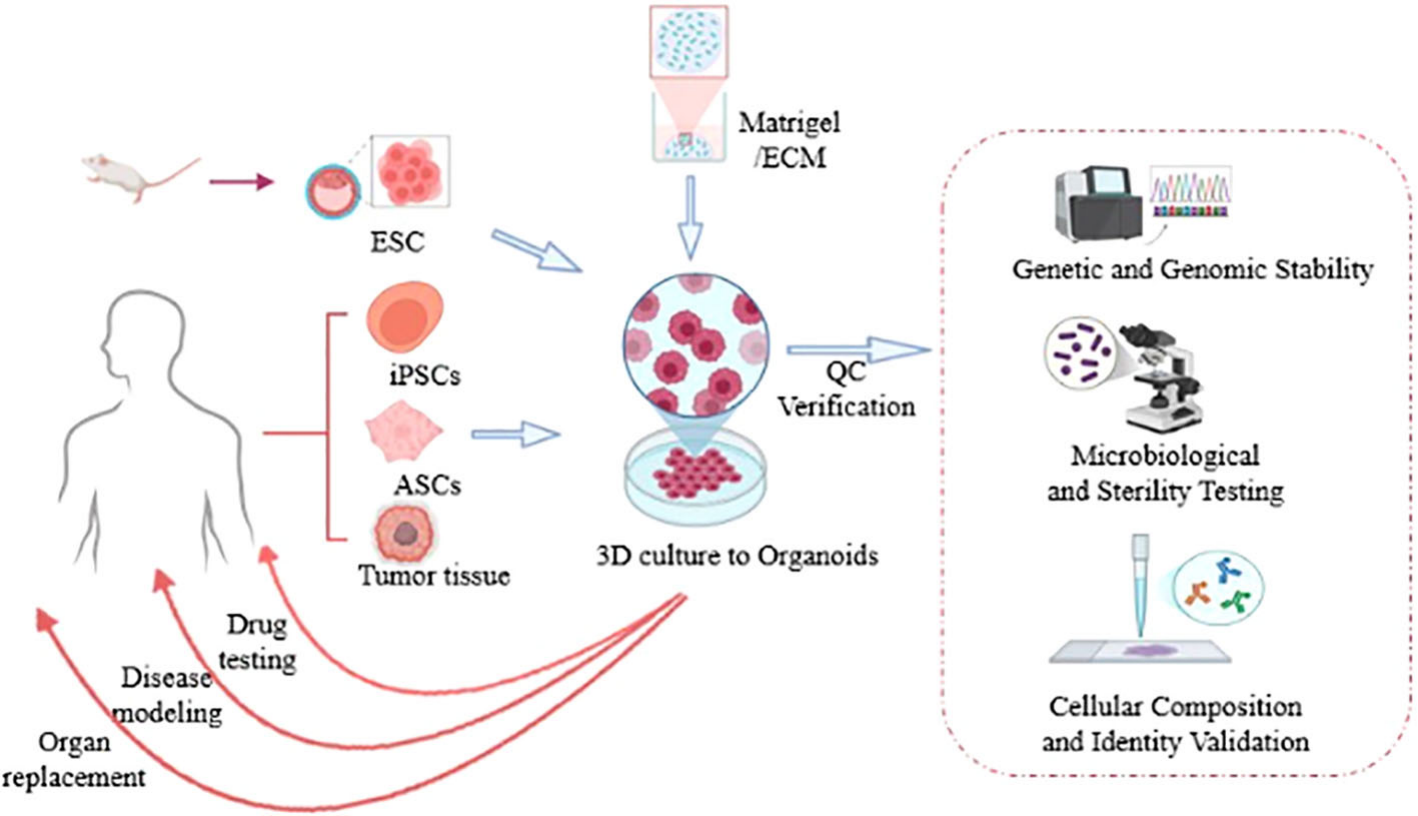
Human tissue samples are isolated, reprogrammed into induced pluripotent stem cells (iPSCs), and subsequently embedded in 3D culture matrices (such as Matrigel) to generate organoids. Following stabilization, these organoids can facilitate the addressing of clinical challenges including disease modeling, drug testing, and organ replacement [Created in http://BioRender.Com].

Organoid-on-a-Chip (OoC), a novel *in vitro* model, utilizes microfluidic chip systems as carrier platforms to construct three - dimensional tissue - like structures. It integrates principles of tissue engineering, microfluidics, and cell biology to establish miniature *in vitro* organ models that mimic the physiological functions and pathological states of organs (5). By addressing the limitations of traditional 2D cell culture models in simulating intercellular interactions and the dynamic changes of the Tumor Microenvironment (TME), OoC provides more authentic experimental data. It offers a more precise platform for exploring tumor biology (6). In recent years, this technology has demonstrated significant potential in cancer therapy research, providing new insights and approaches to address challenges in cancer treatment (**Figure 1**).

## Development of Organoid-on-a-Chip technology

Since the Dutch scientist Hans Clevers and his team first used mouse intestinal adult stem cells to successfully cultivate intestinal organoids with a crypt - villus structure, laying the foundation for organoid technology development (7, 8), OoC technology has experienced three stages of development: the basic research stage focusing on static 3D organoid culture and initially exploring microfluidic technology (2009 – 2015); the multi - organ coupling stage achieving vascularization, multi - organ interaction, and patient - specific modeling (2016 – 2020); and the clinical translation stage entering clinical trials under FDA Act 2.0 (from 2021 to present).

OoC technology has evolved from simple cell aggregates to in - depth research on 3D structures with functional vascular networks (5, 9, 10). The integration of 3D *in vitro* models with Multi - Organ - on - a - Chip (MOC) technology has greatly enhanced the in - vitro drug evaluation level (11). By connecting multiple organ - type models to study the crosstalk between different organs, it can better assess drug safety and efficacy than single - culture models. Organoid research has expanded from the intestine to encompass more complex organs such as the liver, pancreas, and lungs. However, it remains largely confined to culturing single tissue types isolated from within these organs and lacks the ability to recapitulate the dynamic microenvironment found within intact organs. Researchers are actively pursuing strategies to address this fundamental limitation.

Patient - Derived Xenografts (PDX) and Genetically Engineered Mouse Models (GEMM) essentially reflect the unique properties of 3D tumor tissues, compensating for the shortcomings of traditional 2D in - vitro cell cultures that fail to maintain the original tumor morphology and polarity. They provide a more systematic analysis of tumor occurrence, progression, and therapeutic responses. 3D spheroid tumor models can closely replicate in - vivo tumor characteristics, bridging the gap between 2D cell cultures and living tissues and offering more realistic information on tumor internal structure, multicellular composition, and dynamic interactions between cancer cells and the microenvironment. Patient-derived tumor organoids (PDOs) retain key histopathological, genetic, and phenotypic features of the parent tumor with higher fidelity and preserve cancer cell heterogeneity to a greater extent compared to 2D cell lines and PDX models. This superior recapitulation, combined with their favorable balance of success rate and reduced maintenance costs, makes PDOs particularly suitable for large-scale drug screening studies (12). OoC technology models and analyzes diverse pathological states of human organs, drug screening, and therapeutic testing. It enables the creation of patient-specific disease models, presenting unique opportunities to advance inhalation toxicology and drug development by providing a novel, highly biomimetic tool with reduced reliance on animal models (13). Organoid biobanks with associated genomic data offer useful resources for studying cancer cell biology and precision cancer therapy (14, 15). Although tumor organoids have revolutionized cancer research by capturing the cellular structure and behavior of real tumors *in vitro*, their lack of a functional vascular system has hindered their attainment of full physiological capacity. A recently proposed innovative vascularized patient - derived tumor organoid chip, featuring a stratified, tumor - specific microvascular system, provides a versatile platform for exploring tumor vascular dynamics and anti - angiogenic drug efficacy (16).

Technological advances have not only promoted in - depth research on OoC but also driven its applications in drug development and precision medicine (12, 17). In 2013, Science magazine recognized OoC technology as one of the top ten scientific breakthroughs of the year, acknowledging its potential in disease modeling (18). The initial integration of organoids with microfluidic technology enabled the transition from static 3D culture to dynamic microenvironment simulation. In 2017, Nature Methods selected OoC technology as the Method of the Year (19) recognizing its potential in simulating human complex physiological and pathological aspects, especially in drug screening and disease modeling (17). In 2022, the enactment of the FDA Modernization Act 2.0 (20) marked a pivotal regulatory advancement. This legislation sanctioned the first investigational new drug to enter clinical trials based exclusively on preclinical data generated from OoC studies. It eliminated the mandatory animal testing requirement in drug development and established a framework for alternative testing methodologies (21). These provisions accelerate the development of alternative models such as OoC platforms and significantly advance the application of OoC technology in innovative drug discovery (20, 22). For example, PDOs have recently become powerful preclinical models. Georgios Vlachogiannis’s team achieved an 87% accuracy in predicting colorectal cancer drug responses (23).

Currently, OoC technology has made a huge leap from simplicity to complexity, from cells to organs, and from structural simulation to physiological function recreation. Its application scenarios are continuously expanding, covering drug screening, mechanism of action, toxicity studies, disease modeling, precision medicine, and identification of biomarkers and novel toxicity endpoints (6, 24, 25), making it an indispensable tool in biomedical research and drug development. However, the debate over its replacement of animal models continues. Supporters highlight the ethical and cost advantages of OoC (26) while critics question its inability to simulate systemic immune responses (27). Future resolution of this controversy will require technological iteration and policy coordination.

## Applications of Organoid-on-a-Chip in cancer therapy

OoC technology can construct miniaturized organ models with certain physiological functions *in vitro*, mimic cell - cell interactions, and reproduce the dynamic changes of the tumor microenvironment. It has shown significant advantages in exploring tumor mechanisms, drug screening, and disease model establishment (11).

### Research on tumor biological mechanisms

As OoC technology has advanced in simulating tumor microenvironments, it has become more refined in tumor biology research, primarily through two approaches: extrusion-based or photo-crosslinking-based bioprinting using bioinks to fabricate organoids (28) and integrating microfluidic systems into chips (29). Despite partially replicating the tumor microenvironment, both have flaws (**Table 1**). Moreover, integrating complex vascular networks and immune cells with organoids remains a key challenge.

**Table 1:** Technological comparison and limitations.

Technology	Full name & core mechanism	Key advantages	Major limitations	Primary application scenarios
3D-Bioprinted Organoids	Extrusion-based 3D bioprinting of stem cell-laden bioinks	• High structural complexity & anatomical fidelity • Excellent batch reproducibility • Customizable architecture	• Extremely high production cost • Low throughput capacity • Limited vascularization	• Static drug permeability assays • Developmental biology models • Tissue morphogenesis studies
Microfluidic Organ-Chips	Polydimethylsiloxane (PDMS) chips with perfusable microchannels	• Dynamic fluid shear stress simulation • Multi-tissue interface modeling • Real-time imaging compatibility	• Poor immune cell compatibility • Material-dependent drug absorption • Scaling limitations	• Cancer metastasis mechanisms • Nanoparticle drug delivery testing • Vascular barrier function analysis
PDO Models	Patient-derived organoids from tumor biopsies	• Preserves patient-specific genetic heterogeneity • Maintains tumor microenvironment features • High clinical relevance	• Prolonged culture duration (4 – 8 weeks) • Variable success rates • Limited immune component retention	• Personalized therapy screening • Drug resistance profiling • Cancer biomarker discovery

Lai et al. established a co-culture system of fibroblasts with pancreatic tumor organoids, which significantly enhanced collagen deposition and tissue stiffness, thereby recapitulating key aspects of the complex *in vivo* PDAC microenvironment. This engineered 3D vascularized model consequently provided a superior platform for simulating *in vivo* drug transport and distribution. Furthermore, the model revealed differential drug response profiles between direct static administration and perfusion-based vascular delivery, highlighting the critical role of vascular dynamics in therapeutic efficacy (30). Wang Qi’s team at Dalian Medical University Affiliated Hospital successfully simulated lung cancer brain metastasis by constructing upstream “lung” and downstream “brain” units. They discovered that during brain metastasis, intrinsic cellular changes are the primary cause of drug resistance. PC9 - Br cells develop resistance to chemotherapy and EGFR - TKIs through enhanced GSH metabolism, upregulated ALDHs, and inactivated EGFR, which may become key targets for future drug development (31).

Lee’s team employed a bone - on - a - chip model to study breast cancer bone metastasis and revealed that in osteoporotic conditions, increased vascular permeability and reduced mineralization promote bone metastasis (32). Similarly, Christine Trinkle’s team utilized a bone metastasis model and found that in bone microenvironments containing osteoblasts, the extravasation rate of breast cancer cells is significantly increased. The CXCL5 signal enhances tumor cell migration distance, while CXCR2 signal - blocking antibodies decrease extravasation (6). Tang et al. demonstrated that colorectal cancer organoids maintain fidelity to the Wnt/β-catenin signaling expression levels of the original tumor even during long-term culture. This observation suggests that this pathway likely underpins the preservation of cancer stem cell properties and contributes to drug resistance. Furthermore, their findings show that HDAC inhibitors suppress Wnt/β-catenin signaling and significantly reduce cell viability, indirectly suggesting that downregulation of the Wnt/β-catenin pathway may be associated with heightened drug sensitivity (22).

Moorman’s team utilized patient - derived organoid models to study colorectal cancer metastasis and found that phenotypic plasticity is a key mechanism for colorectal cancer metastasis and therapeutic resistance. Tumor cells adapt to environmental stress by dynamically switching states, such as the fetal - like intermediate state, which is a conserved regenerative intermediate state. Its high expression is associated with poor prognosis, suggesting that targeting this state may improve treatment outcomes (25). Ma et al. successfully established an organoid line from tumor cells isolated from a patient’s primary signet-ring cell carcinoma (SRCC) of the colon. Screening this model against an 88-compound library *in vitro* revealed a JAK2 gene mutation, suggesting JAK2 as a potential therapeutic target in colorectal SRCC (33). Furthermore, the study demonstrated that these tumors utilize γ-aminobutyric acid (GABA) as an alternative energy source, enhancing their invasive capacity and conferring a survival advantage (16, 33, 34). This finding not only deepens our understanding of metabolic mechanisms in colorectal cancer but also provides crucial insights for developing novel therapeutic strategies. For instance, interventions targeting the GABA signaling pathway or mechanosensitive pathways may emerge as significant components of future personalized medicine approaches, offering potential new treatments for patients with refractory KRAS-mutant colorectal cancer (35).

Jaehun et al. successfully developed the CATOC system, which recapitulates key features of the *in vivo* tumor microenvironment, including vascular-tumor interactions. By separately developing vascular and tumor modules, this approach minimizes molecular crosstalk, thereby enhancing system stability and reliability (36). Additionally, Yang et al. engineered a vascularized patient-derived tumor OoC platform featuring hierarchical, tumor-specific microvascular networks. Using this model, they discovered that tumor cells with high metastatic potential promote angiogenesis via the Notch signaling pathway and exhibit vasculotropism. Intervention studies with VEGFR2 inhibitors (e.g., apatinib/Apatinib) validated the platform’s utility for evaluating anti-angiogenic therapeutics (16).

These findings collectively demonstrate that OoC platforms effectively recapitulate organ-specific microphysiological environments. These systems are capable of modeling cell-autonomous responses, local tissue microenvironment interactions, absorption, distribution, metabolism, excretion (ADME) processes, and immune system engagement. Consequently, OoC technology serves as a more physiologically relevant platform for *in vitro* screening and a powerful tool for mechanistic investigations. It exhibits a complementary nature with computational models, animal models (particularly those with high translational value), and early-stage clinical research, collectively advancing more accurate and human-relevant drug development and safety assessment.

### Drug screening and clinical translation

The conventional drug discovery pipeline typically initiates with high-throughput screening conducted in 2D cell cultures. This is followed by critical preclinical *in vivo* evaluation using animal models to assess efficacy and safety before advancing to human clinical trials. While these methods can assess drug efficacy and toxicity to some extent, they have notable limitations. Firstly, 2D cell cultures fail to accurately reflect the complexity of the tumor microenvironment, leading to inaccurate predictions of drug responses. Secondly, although animal models are more physiologically similar to humans, species differences often result in disparate drug responses between animals and humans, causing high clinical trial failure rates. Moreover, the prohibitive costs and prolonged culture durations of traditional methods compromise the efficiency and feasibility of drug screening. Consequently, there is an urgent need for higher-throughput, physiologically relevant alternative models to improve success rates and streamline drug development (37).

OoC technology, which combines the advantages of organoids and microfluidic chips, can reconstruct the tumor microenvironment *in vitro*, offering a more authentic platform for testing drug responses (26), at the early stages of drug development, OoC technology can facilitate the identification of drug targets and streamline the drug screening process (38). By preserving the tissue structure and genetic characteristics of patient tumors, organoid chips enable more personalized and precise drug screening (2, 15, 39). Compared to traditional methods, OoC shows higher physiological relevance and lower costs, enabling high - throughput screening in a shorter time, for example, when integrated with the OrBITS high-throughput expansion technology, it can propel the application of PDOs in drug development, therapeutic screening, and personalized treatment guidance (26, 39). Additionally, it allows real - time monitoring of drug effects on cells, providing dynamic data that is unattainable in static cultures (15, 39). OoC has two major applications in drug development: high - throughput drug screening and analysis of drug - resistance mechanisms (26).

Pioneering research by Tang et al. systematically mapped the global transcriptional response landscape of colorectal cancer organoids to 36 therapeutic agents, identifying five universal drug-response patterns: differentiation induction, growth arrest, metabolic suppression, immune activation, and cell cycle blockade. This framework provides molecular signatures for subsequent mechanistic investigations and combinatorial therapy design. Notably, 34 clinically approved or trial-stage drugs were validated for the first time to exhibit significant tumor-suppressive activity in organoids (22). Separately, Minsuh’s team established 80 lung cancer organoids (LCOs) from five histopathological subtypes (adenocarcinoma, squamous cell carcinoma, small cell lung cancer, etc.), which recapitulated the histological and genomic features of primary tumors while preserving tumor heterogeneity. These LCOs demonstrate clinical predictive validity for drug responses: BRCA2-mutant LCOs exhibited sensitivity to olaparib, while EGFR-mutant LCOs showed differential responses to erlotinib contingent upon MET amplification status (40). Regmi’s team designed a droplet microfluidic system for high - throughput drug testing at the single - cell level. They tested the dose - dependent killing of doxorubicin on breast cancer organoids and evaluated the efficacy of tyrosine kinase inhibitors in lung cancer chips (26). Bryan A utilized a microfluidic lung cancer chip system to summarize in - situ cancer growth, treatment responses, and tumor dormancy, finding that EGFR - Ics are currently the preferred treatment for NSCLC (13).

Haque et al. engineered a tumor-on-a-chip platform that recapitulates the pancreatic ductal adenocarcinoma (PDAC) tumor microenvironment (TME). This system maintained PDO functionality and viability long-term, demonstrating that pancreatic stellate cells (PSCs) and U937 monocytes significantly enhance PDO growth and invasiveness. These findings validate the critical role of TME in tumor progression and reveal that stromal-targeting agents (against PSCs and macrophages) potentiate chemotherapeutic efficacy against cancer cells, providing novel strategies for precision therapy in pancreatic cancer (41).Jang’s team devised a microfluidics-based 3D microtumor model to investigate gastric cancer cell behavior and drug resistance. This platform better mimics *in vivo* conditions, elucidating the pivotal role of epithelial-mesenchymal transition (EMT) in tumor progression and chemoresistance. While certain limitations persist, the study establishes a valuable *in vitro* platform for gastric cancer research and provides experimental foundations for developing novel therapeutic approaches (42).Rusyn et al. developed a three-dimensional micro-physiological system (MPS) emulating *in vivo* vascular networks and TME, overcoming constraints of conventional 2D cultures. This integrated platform enables concurrent assessment of drug effects on endothelial and tumor cells, offering comprehensive pharmacological evaluation (43)Zhao’s group established a SARS-CoV-2 infection model using human organoids, creating a new toolset for investigating viral tissue tropism, replication mechanisms, and host-virus interplay. The model uncovered direct SARS-CoV-2-induced impairment of cholangiocyte function, providing mechanistic insights into COVID - 19-associated liver injury pathogenesis (44).

In summary, the organoid - on - a - chip platform provides an ideal testing environment and is emerging as an excellent preclinical drug development platform.

### Personalized and precision medicine

As genomics, proteomics, and metabolomics have advanced in recent years, personalized medicine has found increasing applications, particularly in oncology (45). By analyzing the molecular features of patients’ tumors, physicians can select the most effective treatments, improving patients’ survival rates and quality of life (46). OoC technology, which combines organoid culture and microfluidics, can simulate patient-specific tumor microenvironments *in vitro*. Its advantage lies in preserving tumor heterogeneity and biological characteristics, strongly supporting personalized treatment (38). Researchers can culture patient-derived tumor cells into organoids, screen drugs on microfluidic chips, and monitor tumor responses to different drugs in real time. This improves drug screening efficiency and enables patient-tailored treatment plans (2, 15, 39). In a pioneering study, Hua et al. conducted the first systematic evaluation of PDOs for predicting neoadjuvant chemoradiotherapy responses in locally advanced rectal cancer patients. This work establishes a novel model for personalized and precision medicine, while validating PDOs’ predictive capacity through large-scale clinical specimens. The findings provide compelling evidence to support clinical implementation (46).

OoC technology has also found applications in personalized medicine through PDO and CRISPR-edited models (47). Testing drug efficacy on chips helps accurately identify the best treatment for each patient, improving success rates and reducing side effects. Wang et al. established patient-derived cartilage organoids (PDCOs) that enable precise investigation of joint disorder pathophysiology (e.g., osteoarthritis). This model recapitulates cartilage microphysiology, addressing the critical gap in high-fidelity articular disease modeling. By generating organoids from patients’ own cells, it facilitates development of patient-tailored therapeutic regimens, enhancing treatment efficacy while mitigating adverse reaction risks (48). Tu’s team pioneered a novel strategy for generating human blastoids via a 3D culture system that mimics early embryonic development. This breakthrough establishes an ethical *in vitro* model for human embryogenesis research, creating blastoids independent of exogenous genetic manipulation (e.g., OSKM introduction) and circumventing genome editing-associated concerns (49). While the technology has made significant contributions to personalized and precision medicine, technical limitations must be acknowledged, such as the 21-day PDO culture cycle, which may not meet urgent clinical needs (14), and potential CRISPR off-target effects, which require single-cell sequencing validation (50).

In summary, OoC technology is becoming a key oncology research tool, particularly in simulating complex tumor microenvironments, conducting efficient drug screenings, and promoting personalized treatment. As technology advances, its applications are expected to expand, with improved model accuracy and functionality. Looking ahead, with more research and development, this technology may lead to significant global changes in cancer treatment, offering new hope to patients.

## Discussion

OoC technology provides a robust platform for predicting *in vivo* responses by recapitulating critical tissue microarchitecture and microenvironmental cues at cellular-autonomous and local tissue reaction levels. It further demonstrates considerable promise in modeling absorption, distribution, metabolism, excretion (particularly metabolism), and local immuno-inflammatory processes. Multi-organ chips (MOCs) represent a pivotal developmental direction for investigating inter-organ crosstalk and distal toxicity mechanisms. OoC technology incorporates multiple cell types to assemble functional “micro-organs,” while integrating organoid biology with tissue engineering strategies to better emulate organogenesis processes and physiopathological conditions (48, 51),, has a clear advantage over traditional models. Its self-assembly into 3D structures allows a more authentic reproduction of *in vivo* organ development and partial specific functions. Additionally, organoids maintain genomic stability during expansion, offering a more reliable experimental platform. PDOs are creating new paths for treatment optimization, precise disease modeling, mechanistic studies, drug screening, and personalized therapy strategies. This technology, a blend of advanced methods, is transforming healthcare and improving patient outcomes (49, 52).

Despite demonstrating substantial potential, current OoC systems remain inadequate in recapitulating highly integrated *in vivo* neuro-endocrine-immune regulatory networks. They fail to capture long-term adaptive changes and complex emergent properties, lack precision in reflecting interindividual heterogeneity, and critically lack robust quantitative extrapolation frameworks to translate *in vitro* data into clinically actionable predictors (e.g., therapeutic safety margins). These limitations present persistent challenges for practical implementation.

Standardization gaps in OoC technology critically impede clinical translation. Current organoid cultures exhibit unstable quality control with a notable absence of unified benchmarking criteria (4, 50–54). The technical feasibility and regulatory applicability of interlaboratory standardization remain contentious, as deploying OoC platforms for regulatory purposes necessitates meticulously standardized protocols to ensure reproducible performance metrics across testing facilities (20, 55, 56).

. For instance, inter-laboratory variation in organoid viability testing in China reaches 40% (50). The EU’s “Organoid” initiative has enhanced data comparability by implementing ISO standards like >80% cell viability and <5% genomic instability (20, 55, 56). Large-scale, homogeneous, standardized organoid culture is vital for technology adoption (50, 51). Stricter standard operating procedures and technical specifications are needed to ensure research consistency and accelerate implementation (57).

Cost is another barrier. High-precision microfluidics and complex biomaterials make manufacturing and maintaining OoC expensive, hindering its widespread use in drug screening and clinical applications. Cost reduction and improved cost-effectiveness are critical for future development.

OoC technology involves stem cell use, which has long been ethically controversial (13, 57). Though these models offer new avenues for disease research and therapy, they raise concerns about stem cell sources, usage, and ethical implications. Embryonic stem cell (ESC)-derived organoids, which closely mimic native tissues in function, are strictly restricted in some countries like Germany due to the ethical issue of embryo destruction (58). Induced pluripotent stem cells (iPSCs), on the other hand, avoid some ethical issues but have low reprogramming efficiency (<20%) and potential tumorigenic risks (53). Moving forward, there is a need to promote the use of adult stem cells (such as intestinal Lgr5+ stem cells) as an alternative. Addressing these ethical challenges is crucial for the regulation and legalization of the field. While international organizations and major developed countries have taken multiple steps in the ethical governance of organoid research, China still lacks specific legislation and policies in this area.

Animal models remain central to preclinical research due to their superior simulation of tumor growth, metastasis, and microenvironment interactions compared to organoids and OoC. Though OoC can reduce animal use, animal models are still essential. Future improvements like 3D bioprinting and multi-tissue integrated chips, guided by the “3R principle” (reduction, refinement, replacement), could gradually replace animal experiments (27).

Current research gaps in OoC technology encompass long-term vascularized organoid stability, physiological relevance of multi-organ chips (MOCs), and clinical translation efficiency. Technical challenges include achieving organotypic maturation, reconstructing biomimetic extracellular matrices (ECM), and advancing device fabrication methodologies. Future development priorities focus on vascularization, immune system integration, and system-level emulation to better recapitulate human organ complexity (11, 59).

Critically, functional vascularization is indispensable for organoid growth, development, and physiological functionality. Vascular deficiency constrains organoid scalability and physiological fidelity. Co-culturing vascularized organoids with target tissues enables efficient neovascularization, offering novel conceptual frameworks for precision medicine and disease modeling (23). As research deepens and technology advances, organoid models will improve, potentially leading to new models and tools. They will play a growing role in translational medicine and individualized treatment. Furthermore, applications of OoC in basic biology, drug toxicity testing, and preclinical trials are laying the foundation for advanced technologies like human organ biomimetic chips (41, 51).

## Conclusion and outlook

The core value of OoC technology lies in its innovative integration of microfluidic engineering and biomaterials science, which significantly enhances the biomimicry (e.g., simulating physical microenvironments, multi-tissue interfaces) and individualization potential (e.g., patient-derived models) of *in vitro* systems. This advancement establishes OoCs as transformative platforms for disease modeling (particularly in deciphering tumor heterogeneity and complex tumor microenvironments (TMEs)), drug development (improving preclinical predictive accuracy), precision medicine, and regenerative medicine.

However, realizing the full potential of OoC technology necessitates overcoming several profound technical bottlenecks. The core challenges reside in augmenting the physiological complexity and predictive power of these models:

First, effective integration of functional vascular networks and dynamic immune microenvironments (e.g., T cell infiltration, immune checkpoints) remains a significant engineering hurdle, constraining the accuracy of OoCs in simulating drug delivery and immunotherapy responses. Second, achieving higher cellular functional maturity and precise subtype specificity (e.g., specific neuronal subtypes or cancer stem cell subpopulations) demands advanced bioengineering strategies to overcome prevalent issues like cellular immaturity and insufficient heterogeneity in current models. Third, while simulating multi-organ system (MPS) interactions is a key aspiration, balancing biomimicry, system controllability, and high-throughput screening capability presents a formidable challenge.
