# Correction: Application and development of Organ-on-a-Chip technology in cancer therapy

**DOI:** 10.3389/fonc.2025.1703677

**Published:** 2025-09-24

**Authors:** Ling xiao Wang, Shu ling Liu, Ning Wu

**Affiliations:** ^1^ School of Gongli Hospital Medical Technology, University of Shanghai for Science and Technology, Shanghai, China; ^2^ Shanghai Pudong New Area Gongli Hospital, Shanghai, China; ^3^ Department of Oncology, Shanghai Pudong New Area Gongli Hospital, Shanghai, China

**Keywords:** *in vitro* model, biomedical engineering, drug screening, personalized medicine, organ-on-a-chip, cancer therapy

A correction has been made to the section “**Applications of Organoid-on-a-Chip in cancer therapy**” sub-section “*Drug screening and clinical translation*”, 3^rd^ paragraph. The citation number was erroneously given as 25 (Moorman et al.) instead it should have been 26 (Regmi et al.). The corrected sentence appears below: “Regmi’s team designed a droplet microfluidic system for high - throughput drug testing at the single -cell level. They tested the dose - dependent killing of doxorubicin on breast cancer organoids and evaluated the efficacy of tyrosine kinase inhibitors in lung cancer chips (26).”

A correction has been made to the section “**Applications of Organoid-on-a-Chip in cancer therapy**”, sub-section “*Research on tumor biological mechanisms*” 3rd paragraph. The sentence and citation to reference 33 (Gjerdrum et al.) was erroneous. The corrected sentence and citation appears below:

“Similarly, Christine Trinkle’s team utilized a bone metastasis model and found that in bone microenvironments containing osteoblasts, the extravasation rate of breast cancer cells is significantly increased. The CXCL5 signal enhances tumor cell migration distance, while CXCR2 signal - blocking antibodies decrease extravasation (6).”

A correction has been made to the section “**Applications of Organoid-on-a-Chip in cancer therapy**”, sub-section “*Research on tumor biological mechanisms*” 4th paragraph. The reference details of reference 35 were given as “Takebe, T., & Wells, J. M. (2019). Organoids by design. Science (New York, N.Y.), 364(6444), 956–959. https://doi.org/10.1126/science.aaw7567”.

The correct reference details are:

“35. Strelez C, Perez R, Chlystek JS, Cherry C, Yoon AY, Halliday B, et al. Integration of Patient-Derived Organoids and Organ-on-Chip Systems: Investigating Colorectal Cancer Invasion within the Mechanical and GABAergic Tumor Microenvironment. bioRxiv [Preprint]. 2023 Sep 17:2023.09.14.557797. doi: 10.1101/2023.09.14.557797.”

An incorrect **Funding** statement was provided. The correct funder and grant number is: “Shanghai Pudong New District Science and Technology Commission, Grant Number PKJ2023-Y25”.

A correction has been made to the section “**Applications of Organoid-on-a-Chip in Cancer Therapy**”, sub-section “*Research on Tumor Biological Mechanisms*”, Paragraph 1. There was an error in the text referencing Table 1. The paragraph has been corrected as follows:

“As OoC technology has advanced in simulating tumor microenvironments, it has become more refined in tumor biology research, primarily through two approaches: extrusion-based or photo-crosslinking-based bioprinting using bioinks to fabricate organoids (28) and integrating microfluidic systems into chips (29). Despite partially replicating the tumor microenvironment, both have flaws (Table 1). Moreover, integrating complex vascular networks and immune cells with organoids remains a key challenge.”

The original version of this article has been updated.

